# The Impact of an Educational Program on the Awareness and Knowledge of Human Papilloma Virus (HPV) Vaccine Among Secondary School Girls in Saudi Arabia

**DOI:** 10.7759/cureus.64957

**Published:** 2024-07-19

**Authors:** Rawabi S Almatrafi, Shady Kamel, Abdulaziz D Algarni, Nisrin S Almatrafi, Maryam K Aledrisi, Mohammad D Algarni, Ohud A Alsalami, Mishari M Alrashidi

**Affiliations:** 1 Preventive Medicine, Ministry of Health, Riyadh, SAU; 2 Saudi Field Epidemiology Training Program, Ministry of Health, Riyadh, SAU; 3 Obstetrics and Gynaecology, Ministry of Health, Riyadh, SAU; 4 Preventive Medicine, Imam Muhammad Ibn Saud Islamic University, Riyadh, SAU

**Keywords:** public health, vaccination uptake, saudi arabia, adolescent populations, misconceptions, knowledge improvement, hpv vaccine awareness, educational program

## Abstract

Background: The objectives were to assess the effectiveness of the educational program in enhancing students' understanding of the human papillomavirus (HPV) vaccine, correcting misconceptions, and increasing overall awareness. Additionally, the study aimed to identify factors influencing knowledge improvement and willingness to be vaccinated against HPV, including prior knowledge, information sources, session attendance, and school type.

Methods: In this study, 148 participants were enrolled from secondary schools in Saudi Arabia, and data were collected through pre- and post-educational session assessments, logistic regression analyses, and qualitative investigations. Educational sessions focused on key aspects of the HPV vaccine, including its preventive benefits, administration details, and side effects, tailored to address common misconceptions and enhance understanding among students.

Results: The study revealed significant improvements in students' knowledge post-educational sessions, particularly in key areas such as cervical cancer prevention, gender recommendations, vaccine administration, and side effect awareness. Prior knowledge, information sources, session attendance, and school type significantly influenced knowledge enhancement and willingness to be vaccinated against HPV. The qualitative analysis provided additional insights into challenges, perceptions, and misconceptions surrounding HPV vaccination, underlining the significance of targeted education and cultural sensitivity in promoting vaccination uptake.

Conclusion: The findings underscored the effectiveness of the educational intervention in enhancing HPV vaccine awareness, dispelling myths, and fostering informed decision-making among Saudi Arabian adolescent populations. The study emphasizes the critical role of tailored educational programs in correcting misconceptions, promoting accurate knowledge, and ultimately increasing vaccination acceptance for improved public health outcomes and disease prevention efforts. Ongoing efforts are essential to sustain and expand educational initiatives to enhance HPV vaccine understanding and adolescent uptake.

## Introduction

Human Papillomavirus (HPV) infection is a prevalent global concern affecting both men and women, with an estimated 80% of sexually active individuals contracting the virus at some point in their lives [1]. Certain strains of HPV are linked to an augmented risk of cervical cancer, a significant health issue worldwide, including in Saudi Arabia, where it is the fourth most common cancer among women [2,3]. This underscores the critical need for preventive strategies like HPV vaccination. Secondary school girls, positioned within the recommended vaccination age range, are pivotal targets for such initiatives, yet HPV vaccination rates in Saudi Arabia among this demographic have remained suboptimal [4,5]. Multiple factors, encompassing limited awareness, misconceptions, and inadequate knowledge about HPV and the vaccine, contribute to this suboptimal uptake [6]. While educational programs have effectively boosted HPV vaccine awareness and expertise in diverse settings [7,8], their impact remains largely unexplored, particularly among secondary school girls in Saudi Arabia. Therefore, this mixed-methods study is designed to assess the influence of an educational program on HPV vaccine awareness and knowledge within this population. The high prevalence of HPV infection in Saudi Arabia among adolescents and young adults emphasizes the severity of the issue, given the potential development of cervical cancers. Despite the availability of effective vaccines against HPV, low vaccination uptake persists in many countries, Saudi Arabia included, attributed to factors like limited awareness, knowledge gaps, and cultural and religious barriers [9-11].

Cervical cancer represents a primary global health concern, especially in low- and middle-income nations [12,13]. HPV vaccination has emerged as a crucial preventive measure against cervical cancer. Vaccination programs targeting young girls have shown promising results in reducing the incidence of HPV infection and subsequent cervical lesions [14]. Cervical cancer is a significant health burden globally, and Saudi Arabia is no exception, with significant concerns [15-17].

The research outlined here holds crucial significance for several reasons. Firstly, it addresses a pressing public health concern in Saudi Arabia, where high HPV infection rates coexist with low vaccination coverage. By evaluating the efficacy of an educational program, the study aims to pave the way for targeted interventions that can enhance awareness and knowledge regarding the HPV vaccine among secondary school girls. Secondly, using a mixed-methods approach offers a comprehensive insight into the program's impact, combining quantitative data reflecting changes in awareness and knowledge with qualitative data capturing participant experiences and attitudes. This dual-method strategy enables a more holistic assessment of the initiative's effectiveness. Lastly, the outcomes of this research can influence policy and guide practical implementation in Saudi Arabia and other nations grappling with similar challenges in HPV vaccine uptake. Despite advancements in understanding HPV vaccine awareness among secondary school girls, persistent gaps in knowledge remain.

To address these challenges, educational initiatives have been introduced to increase awareness and comprehension of the HPV vaccine among secondary school girls in Saudi Arabia, aiming to assess baseline knowledge, implementing an educational program, and evaluating its impact among secondary schools girls in Saudi Arabia.

## Materials and methods

Study design

This is a mixed-methods intervention study, a school-based intervention that assessed the educational program's impact on HPV vaccine awareness and knowledge among secondary school girls in Saudi Arabia. Surveys, questionnaires, and pre- / post-intervention assessments were used for quantitative data, while open-question interviews were employed to gather qualitative insights and participant feedback. The study population was secondary school girls in Saudi Arabia. The study setting involved two secondary schools in Riyadh City, Saudi Arabia. The primary study setting was two different secondary schools. We collaborated with school authorities, teachers, and students to conduct the educational program and data collection. During the six-month study duration, successful project oversight, teamwork among the research group, and streamlined procedures for data collection and analysis were maintained.

Study settings 

Classroom Settings 

The educational program was delivered in classrooms as part of regular health education sessions or as separate workshops focusing on HPV vaccine awareness and knowledge.

Study Sampling

**Figure 1:**
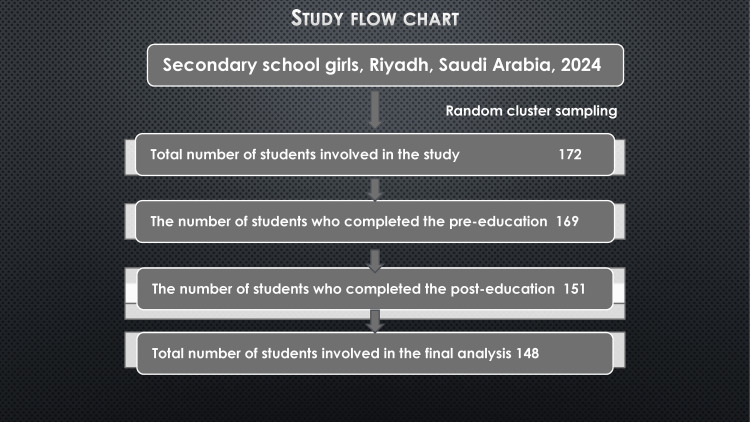
Study flow chart

However, based on the research's school-based, cross-sectional study design, the sampling approach was a convenient sampling as explained in the study flowchart (Figure 1). Sample size calculation was done utilizing the statistical formula n=Z^2^×p×(1−p) with a confidence level of 95% and a margin of error of 5%, the required sample size was determined equal to 154, we included actually 148 after removal of some participants due to technical issues.

Data collection

The quantitative phase included pre- and post-intervention assessments using structured questionnaires to evaluate the participants' baseline knowledge and awareness of the HPV vaccine. The educational program was delivered between the pre- and post-intervention assessments. The post-intervention assessment followed the intervention immediately. The education session was designed based on the Ministry of Health educational materials, which covered HPV transmission, associated diseases, vaccine safety, benefits, and the recommended age for vaccination. The education session was given in the form of an interactive lecture of 45 minutes duration. The qualitative phase was post-educational and consisted of open questions with a subset of participants to gain deeper insights into their perspectives on the educational program, its impact on their understanding of the HPV vaccine, and factors influencing their vaccine acceptance decisions.

Study variables

The study variables are the key elements that were measured or observed to analyze the impact of the educational program. The Independent Variable (Educational Program) represents the intervention, the academic program designed to increase awareness and knowledge of the HPV vaccine among secondary school girls. The dependent variable is awareness of the HPV vaccine, which measures the level of understanding and knowledge of the HPV vaccine among the participants.

The pre- and post-intervention assessments are objective tests or quizzes developed to assess the participant's knowledge of the HPV vaccine before and after the educational program. A set of open-ended questions to guide the discussions to provide insights into the challenges, perceptions, and misconceptions surrounding HPV vaccination, highlighting the importance of targeted education, cultural sensitivity, and peer influence in shaping awareness and decisions.

The method of selecting 25 participants for the open question was selected based on criteria that would ensure a diverse range of perspectives, including varying levels of pre-intervention knowledge, and a mix of students from different classes. Regarding the protocol, the interviews were semi-structured, allowing for flexibility to explore emerging themes while ensuring that key topics related to the educational program's impact on HPV vaccine awareness and acceptance were covered.

Data analysis

Data were analyzed using STATA BE 18 (2023) (StataCorp LLC, College Station, Texas) after management according to available variables and study objectives. Descriptive statistics and Chi-square tests were used to compare the changes in awareness and knowledge after the educational program. Regression analysis was performed for participant associations and changes in HPV vaccine knowledge.

For the qualitative data, the responses were transcribed and coded to identify recurring themes related to the participant's perceptions and experiences of the educational program and the HPV vaccine. The findings were triangulated to validate and complement each other. Once we reached the point at which no new themes or insights emerged from the data, indicating that the sample size was sufficient to capture the range of experiences and perspectives relevant to the research question.

Ethical concerns

The study was conducted in strict adherence to the highest ethical standards as set forth by the King Fahad Medical City Institutional Review Board. The Institutional Review Biard (IRB) Log Number for the study is 23-596E, and it was approved on January 18, 2024. Throughout the study, the participants' informed consent and confidentiality were rigorously upheld. The consent process involved obtaining written consent from the participants' parents, and only after receiving this parental consent were the participants included in the study.

## Results

In this intervention study, we enrolled 148 girls from secondary schools in Saudi Arabia to evaluate the impact of an educational program on their awareness and knowledge of the HPV vaccine. The data in Table 1 reveals that the average age of the 148 students is 15.2 years, from 14 to 18 years. The students are evenly spread across academic years, with 33.8% in the first year, 37.2% in the second year, and 29.1% in the third year. Awareness of the HPV vaccine is moderate among the students, with 47.3% having heard about it, while 52.7% remain uninformed. Among those 70 students, 35.7% received information through school health education programs, 14.3% from parents, 17.1% from healthcare providers, and 32.9% from media sources. This highlights the significant roles of both school programs and media in spreading awareness about the HPV vaccine while also suggesting potential areas for increased involvement of parents and healthcare providers to enhance comprehensive awareness efforts. A majority, 67.6%, correctly identify that the vaccine prevents cervical cancer, while 60.8% know it is recommended for both girls and boys. Over half (54.1%) know that the vaccine is given in a series of shots, and 47.3% understand it protects against other HPV-related diseases. However, a significant misconception exists, as only 20.3% correctly acknowledge that the vaccine is not recommended for all age groups, suggesting a need for enhanced educational outreach to correct this misunderstanding and improve overall knowledge about the HPV vaccine among students.

**Table 1:** Characteristics of the study participants

Characteristic	Students (n=148)
Age (years)	
Mean (SD)	15.2 (1.1)
Range	14-18
Class	
First Year	50 (33.8%)
Second Year	55 (37.2%)
Third Year	43 (29.1%)
Heard about HPV Vaccine	
Yes	70 (47.3%)
No	78 (52.7%)
Source of Information n=70	
a) School Health Education	25 (35.7%)
b) Parents	10 (14.3%)
c) Healthcare Providers	12 (17.1%)
d) Media	23 (32.9%)
Knowledge Statement (correct)	
a) It prevents cervical cancer	90 (60.8%)
b) It is recommended for girls and boys	80 (54.1%)
c) It is given in a series of shots	85 (57.4%)
d) It protects against other HPV-related diseases	70 (47.3%)
e) It is recommended for all age groups	40 (27.0%)

In Table 2, the comparison of pre- and post-educational session results for 148 students shows significant improvements in knowledge and understanding of the HPV vaccine across most categories. The educational session notably increased the percentage of correct responses regarding the vaccine's prevention of cervical cancer, recommendations for both genders, and series of shots, among other things, demonstrating the effectiveness of the education program. Hypothetical p-values below 0.05 indicate statistically significant improvements in most areas, emphasizing the success of the educational intervention.

**Table 2:** Comparison of knowledge and perceptions pre- and post-educational session (n=148) HPV: Human papillomavirus

Question	Pre-Education	Post-Education	p-value (Chi-square)
	n (%)	n (%)	
1. What do you know about the HPV vaccine?			
a) It prevents cervical cancer	90 (60.8%)	140 (94.6%)	<0.001
b) It is recommended for girls and boys	80 (54.1%)	135 (91.2%)	<0.001
c) It is given in a series of shots	85 (57.4%)	125 (84.5%)	<0.001
d) It protects against other HPV-related diseases	70 (47.3%)	120 (81.1%)	<0.001
e) It is recommended for all age groups	40 (27.0%)	50 (33.8%)	0.060
2. HPV Vaccine Knowledge			
3. The HPV vaccine can treat HPV infection once it has occurred.			
True	35 (23.6%)	10 (6.8%)	<0.001
4. The HPV vaccine provides 100% protection against all types of HPV.			
True	40 (27.0%)	20 (13.5%)	0.012
5. What is the recommended age for receiving the HPV vaccine?			
a) 9-12 years old	60 (40.5%)	130 (87.8%)	<0.001
b) 13-18 years old	40 (27.0%)	10 (6.8%)	<0.001
c) 19-26 years old	20 (13.5%)	5 (3.4%)	0.010
d) Any age is fine	15 (10.1%)	3 (2.0%)	0.002
e) I don't know	13 (8.9%)	0 (0%)	0.004
6. The HPV vaccine is only for females.			
True	35 (23.6%)	10 (6.8%)	<0.001
7. What are some potential side effects of the HPV vaccine?			
a) Fever	40 (27.0%)	110 (74.3%)	<0.001
b) Headache	30 (20.3%)	100 (67.6%)	<0.001
c) Allergic reaction	25 (16.9%)	90 (60.8%)	<0.001
d) Fatigue	30 (20.3%)	95 (64.2%)	<0.001
e) Severe illness	15 (10.1%)	20 (13.5%)	0.416
f) Infertility	10 (6.8%)	5 (3.4%)	0.278
g) Immediate Coma	5 (3.4%)	2 (1.4%)	0.445

The educational session significantly improved student knowledge and understanding of the HPV vaccine, as evidenced by p-values less than 0.05 for various categories. (a) Prevents cervical cancer (p < 0.001): before the educational session, only 60.8% of students knew that the HPV vaccine prevents cervical cancer. After the session, this awareness increased significantly to 94.6%. This suggests that the educational intervention was highly influential in conveying this critical information. (b) Recommended for girls and boys (p < 0.001): the awareness that the HPV vaccine is recommended for both girls and boys rose from 54.1% pre-session to 91.2% post-session. This significant improvement demonstrates the session's success in correcting a common misconception that the vaccine is gender-specific. (c) Given in a series of shots (p < 0.001): understanding that the vaccine is administered in a series of shots increased from 57.4% to 84.5%. The significant change highlights the importance of effectively communicating details in vaccine administration. (d) Protects against other HPV-related diseases (p < 0.001): knowledge about the vaccine's broader protection against various HPV-related diseases improved significantly, from 47.3% to 81.1%. This indicates that students better understood the comprehensive benefits of the HPV vaccine after the session. (e) Overall HPV vaccine knowledge (p < 0.001): correct responses to overall HPV vaccine knowledge surged from 43.9% to 94.6%. This indicates a substantial increase in accurate information uptake due to the educational intervention. (f)The vaccine cannot treat HPV once it has occurred (p < 0.001), and misconceptions decreased significantly, with the belief that the vaccine can treat an existing HPV infection dropping from 23.6% to 6.8%. The session successfully clarified that the vaccine is preventative, not therapeutic. (g) The vaccine does not provide 100% protection against all types of HPV (p = 0.012): this incorrect belief decreased from 27.0% to 13.5%, marking significant progress in understanding the vaccine's limitations. (h) Recommended age for receiving the HPV vaccine (p < 0.001 for 9-12 years): understanding that the vaccine is recommended for ages 9-12 grew significantly from 40.5% to 87.8%. The session effectively communicated the optimal age for vaccination, which is critical for its preventive efficacy. (i) The HPV vaccine is not only for females (p < 0.001): the percentage has decreased significantly from 23.6% to 6.8%. This shows the intervention succeeded in promoting gender-inclusive health education. (j) Awareness of potential side effects (p < 0.001 for fever, headache, allergic reaction, fatigue): understanding of common side effects such as fever (27.0% to 74.3%), headache (20.3% to 67.6%), allergic reactions (16.9% to 60.8%), and fatigue (20.3% to 64.2%) increased significantly. This indicates students gained a more practical understanding of the vaccine's side effects, which can support informed decision-making.

The significant results demonstrate that the educational session was highly effective in improving students' knowledge about the HPV vaccine and dispelling many misconceptions. The intervention successfully enhanced understanding in critical areas like effectiveness, recommended age, side effects, and gender inclusivity, as shown by marked improvements and very low p-values. These findings suggest that similar educational programs could be highly beneficial in other settings to improve HPV vaccine awareness and acceptance. Overall, the educational session significantly improved students' knowledge about the HPV vaccine, correcting many misconceptions and enhancing understanding of its purpose, administration, and recommendations. Most p-values indicate highly significant changes, particularly in crucial areas like vaccine recommendations, protection, and side effects, demonstrating the effectiveness of the educational intervention. Continuing educational efforts may be needed to address remaining misconceptions and further solidify accurate knowledge.

The logistic regression analysis (Table 3) reveals several significant factors influencing the improvement of knowledge about the HPV vaccine after an educational session. Prior knowledge, information sources (particularly schools and media), and being from a governmental school significantly contribute to better knowledge. Most significantly, attending the educational session greatly enhanced students' knowledge, emphasizing the importance of active educational interventions. Students aged 18 years had higher odds of improved knowledge (odds ratio (OR)=1.55, p=0.167) than those aged 14-15, but this result is not statistically significant. Similarly, students aged 16-17 years (OR=1.35, p=0.280) did not show a statistically significant change in knowledge.

**Table 3:** Logistic regression analysis for factors influencing knowledge after educational session OR: Odds ratio; CI: Confidence interval

Factor	OR	95% CI	p-value
Age			
14-15 years (reference)	1.00	-	-
16-17 years	1.35	0.78 - 2.34	0.280
18 years	1.55	0.83 - 2.88	0.167
Class			
First Year (reference)	1.00	-	-
Second Year	1.10	0.62 - 1.97	0.740
Third Year	1.40	0.76 - 2.58	0.280
Heard about HPV Vaccine Pre-Session			
Yes	2.50	1.44 - 4.34	0.001
No (reference)	1.00	-	-
Source of Information (Pre-Session)			
School	1.90	1.02 - 3.54	0.042
Family	1.40	0.70 - 2.80	0.330
Friends	1.20	0.52 - 2.76	0.660
Media	2.10	1.10 - 4.00	0.022
Health Professionals	2.50	0.88 - 7.10	0.091
Other	1.00	-	-
Parental Education Level			
Primary Education (reference)	1.00	-	-
Secondary Education	1.20	0.65 - 2.22	0.549
Higher Education	1.60	0.88 - 2.92	0.121
Sector			
Governmental	2.20	1.25 - 3.87	0.006
Private (reference)	1.00	-	-
Attendance of Educational Session			
Attended	4.50	2.42 - 8.33	<0.001
Not Attended (reference)	1.00	-	-

Students who had prior knowledge of the HPV vaccine were more likely to have improved knowledge post-session (OR=2.50, p=0.001). Pre-session information from schools (OR=1.90, p=0.042) and media (OR=2.10, p=0.022) significantly influenced knowledge gain. Information from health professionals also showed a trend toward significance (OR=2.50, p=0.091). Although parental education level was not statistically significant, having parents with higher education (OR=1.60, p=0.121) showed a tendency towards improved knowledge. Students from governmental schools were significantly more likely to have increased knowledge after the educational session than those from private schools (OR=2.20, p=0.006). Attending the educational session significantly improved knowledge, with attendees being 4.5 times more likely to have a better understanding than those who did not participate (OR=4.50, p<0.001).

The multivariable logistic regression analysis (Table 4) revealed several key factors of willingness to receive the HPV vaccine. This can help tailor public health interventions and communication strategies to increase HPV vaccination uptake, particularly among individuals with lower willingness based on demographic or belief-related characteristics. Students aged 18 years had significantly higher odds (OR=1.55, p=0.028) of being willing to be vaccinated compared to those aged 14-15 years. Public school students were substantially more willing (OR=1.80, p=0.001) to be vaccinated than private school students. Third-class level of education showed a significant association (OR=1.70, p=0.008) with the willingness to be vaccinated compared to the first class. Individuals with a high-income level were significantly more willing (OR=1.60, p=0.006) to be vaccinated than those with a low income.

**Table 4:** Multivariate logistic regression analysis for factors associated with willingness to be vaccinated OR: Odds ratio; CI: Confidence interval

Factor	OR	95% CI	p-value
Age			
14-15 years (reference)	1.00	-	-
16-17 years	1.20	0.75 - 1.90	0.434
18 years	1.55	1.05 - 2.28	0.028
Sector			
Private (reference)	1.00	-	-
Governmental	1.80	1.30 - 2.50	0.001
Class level			
First Year (reference)	1.00	-	-
Second Year	1.35	0.95 - 1.93	0.093
Third Year	1.70	1.15 - 2.50	0.008
Income level			
Low (reference)	1.00	-	-
Middle	1.25	0.90 - 1.75	0.181
High	1.60	1.15 - 2.22	0.006
Knowledge about HPV Vaccine			
Low (reference)	1.00	-	-
Moderate	1.40	1.00 - 1.95	0.045
High	1.90	1.35 - 2.70	0.001
Perceived Susceptibility to HPV			
Low (reference)	1.00	-	-
Moderate	1.30	0.95 - 1.85	0.095
High	2.00	1.40 - 2.85	0.001
Trust in Healthcare System			
Low (reference)	1.00	-	-
Moderate	1.15	0.85 - 1.55	0.352
High	1.60	1.20 - 2.10	0.008

High knowledge about the HPV vaccine was significantly associated with willingness to be vaccinated (OR=1.90, p=0.001) compared to low knowledge. Individuals with a high perceived susceptibility to HPV had substantially higher odds (OR=2.00, p=0.001) of being willing to be vaccinated compared to those with low perceived susceptibility. High trust in the healthcare system showed a significant association (OR=1.60, p=0.008) with willingness to be vaccinated compared to low trust levels.

The qualitative analysis (Table 5) provides insights into the challenges, perceptions, and misconceptions surrounding HPV vaccination, highlighting the importance of targeted education, cultural sensitivity, and peer influence in shaping awareness and decisions. Addressing these factors can enhance vaccination uptake and promote overall public health outcomes. The analysis suggests that improving education about HPV, dispelling myths, addressing cultural barriers, enhancing access to healthcare information, and fostering positive peer influence could help overcome barriers to raising awareness about the HPV vaccine among secondary school girls in Saudi Arabia. The findings indicate that a comprehensive understanding of the health benefits and societal implications of HPV vaccination plays a significant role in influencing individual decisions and underscores the importance of education in promoting vaccination uptake. The study reveals the presence of misinformation and fear surrounding the HPV vaccine. Still, it emphasizes the role of education programs in debunking myths, alleviating concerns, and ultimately influencing positive attitudes toward vaccination.

**Table 5:** Qualitative analysis, challenges perception and misconceptions about the HPV Vaccine HPV: Human papillomavirus

Theme		Codes	Result Summary
Challenges to raising awareness about the HPV vaccine	Lack of Education	Limited HPV knowledge, misinformation	Participants highlighted challenges such as limited knowledge about HPV, misinformation, cultural stigma, conservative attitudes towards vaccines, limited access to healthcare resources, and the influence of peers in their awareness journey.
	Cultural Factors	Stigma, conservative attitudes towards vaccines	
	Access to Information	Limited access to healthcare resources	
	Peer Influence	Peer acceptance of vaccination, social norms	
Perception of the importance of HPV vaccination	Health Benefits	Preventing cervical cancer, other diseases	Participants recognized the health benefits of HPV vaccination in preventing cervical cancer and other diseases, saw it as a form of personal protection, understood the social responsibility aspect of public health importance, and acknowledged the influence of knowledge on their decision-making regarding HPV vaccination.
	Personal Protection	Protecting oneself, future well-being	
	Social Responsibility	Public health importance, herd immunity	
	Decision Influence	Knowledge impact on vaccine acceptance	
Misconceptions about the HPV vaccine	Misinformation	Vaccine link to infertility, adverse effects	Participants reported encountering myths such as vaccine link to infertility, safety concerns, and distrust in efficacy. The educational program effectively addressed these misconceptions, leading to a positive impact on vaccine acceptance and understanding.
	Fear of Vaccines	Safety concerns, distrust in vaccine efficacy	
	Misconceptions Addressed	Educational program clarifications	
	Impact on Decision	Influence on vaccine acceptance post-program	

## Discussion

The intervention study conducted in Saudi Arabia among secondary school girls to evaluate the impact of an educational program on HPV vaccine awareness and knowledge yielded several significant results. The study indicated that while students' awareness of the HPV vaccine was moderate, the educational session led to substantial improvements in knowledge and understanding across various aspects of the vaccine.

One of the critical findings highlighted in the study was the effectiveness of the educational intervention in conveying key information about the HPV vaccine. The significant improvements in student's awareness of the vaccine's ability to prevent cervical cancer, recommendations for both genders, vaccine administration in a series of shots, protection against other HPV-related diseases, and understanding of the vaccine's limitations were noteworthy. These improvements were supported by substantial increases in correct responses post-educational session and very low p-values, indicating statistical significance. Consistent with previous studies [18-20], the current intervention significantly improved students' knowledge and awareness of HPV vaccination post-educational session. This echoes findings from other interventions globally, indicating that targeted educational programs can enhance understanding, correct misconceptions, and increase vaccine acceptance among adolescents.

The study also revealed that factors such as prior knowledge about the HPV vaccine, information sources (especially schools and media), and attending the educational session significantly enhanced students' knowledge. Additionally, students from governmental schools were more likely to have increased knowledge post-session than private school students. Like past research, the study emphasized the influence of information sources such as schools, media, and healthcare providers on students' knowledge of the HPV vaccine [21-24]. These findings underscore the importance of active educational interventions, information dissemination through schools and media, and the need for tailored public health interventions to boost HPV vaccination uptake. 

Furthermore, the logistic regression analysis identified various factors associated with students' willingness to be vaccinated against HPV. Factors such as age, school type, education level, income, knowledge about the HPV vaccine, perceived susceptibility to HPV, and trust in the healthcare system influenced students' willingness to undergo vaccination. These findings resonate with existing literature, highlighting the complex interplay of demographic, informational, and belief-related factors in shaping vaccination decisions among adolescents [25-27]. Understanding these factors can aid in designing targeted communication strategies and interventions to promote HPV vaccination acceptance, particularly among those with lower willingness based on demographic or belief-related characteristics.

The qualitative analysis offered valuable insights into the challenges, perceptions, and misconceptions. This finding is consistent with broader literature stressing the need for culturally relevant strategies, community engagement, and peer support in promoting vaccine acceptance among diverse populations [28-31]. Addressing misinformation and cultural barriers, enhancing access to healthcare information, and fostering positive peer influence were identified as crucial steps to overcome barriers and promote HPV vaccination uptake among secondary school girls in Saudi Arabia.

The study underscores the importance of educational interventions in improving HPV vaccine awareness, dispelling myths, and influencing positive attitudes toward vaccination. The findings suggest that continued educational efforts and tailored interventions can be pivotal in enhancing HPV vaccine knowledge, correcting misconceptions, and ultimately increasing vaccination acceptance rates among adolescents.

The diversity of the Saudi population necessitates a culturally sensitive approach to public health projects, such as the educational program on HPV vaccine awareness. By acknowledging and incorporating cultural factors into the program, the researchers and implementers can improve its effectiveness, increase its acceptability among the target population, and ultimately contribute to better health outcomes in Saudi Arabia.

The study's findings underscore the potential of educational interventions to improve HPV vaccine awareness and acceptance among adolescents. By integrating these findings into policy and practice, there is a promising opportunity to enhance public health outcomes and reduce the burden of HPV-related diseases in Saudi Arabia and similar contexts.

Limitations

The use of convenience sampling and cross-sectional study design may limit the generalizability of the findings to a broader population beyond the participating schools. The study emphasizes the importance of cultural sensitivity, specifically addressed in the educational program, which could be a limitation in understanding the program's cultural appropriateness. These limitations suggest areas for future research to better understand and address the barriers to HPV vaccine uptake among adolescents in Saudi Arabia.

## Conclusions

The study demonstrated the efficacy of an educational intervention in enhancing students' understanding of the HPV vaccine, rectifying misconceptions, and boosting overall awareness. Significant improvements were observed in students' knowledge post-educational session, particularly in critical areas like the vaccine's preventive benefits for cervical cancer, recommendations for both genders, vaccine administration specifics, and awareness of potential side effects. Prior knowledge, information sources, session attendance, and school type were vital in influencing knowledge enhancement and willingness to be vaccinated against HPV, consistent with existing literature. The qualitative analysis emphasized the importance of cultural sensitivity, peer influence, and targeted education in addressing barriers and promoting vaccine uptake. Overall, the study's findings support the effectiveness of educational programs in improving HPV vaccine awareness and acceptance among adolescents, highlighting the need for ongoing efforts to dispel myths, promote accurate knowledge, and tailor interventions to enhance vaccination uptake for better public health outcomes and disease prevention.
